# Association Between Food Insecurity and Poor Cardiovascular Health Assessed by the Life’s Essential 8 Metric: A Population-Based Study of Korean Adults

**DOI:** 10.3390/nu17132148

**Published:** 2025-06-27

**Authors:** Seong-Uk Baek, Jin-Ha Yoon

**Affiliations:** 1 Graduate School, Yonsei University College of Medicine, Seoul 03722, Republic of Korea; 2 Department of Preventive Medicine, Yonsei University College of Medicine, Seoul 03722, Republic of Korea; 3 The Institute for Occupational Health, Yonsei University College of Medicine, Seoul 03722, Republic of Korea

**Keywords:** cardiovascular risk factors, cardiovascular disease, food security, health inequality, nutrition

## Abstract

Background/Objectives: Access to nutritious and well-balanced food is essential for well-being. We investigated the relationship between food insecurity (FI) and cardiovascular health (CVH). Methods: This cross-sectional analysis included a nationwide sample consisting of 12,369 Korean adults. The 18-item Household Food Security Survey Module was employed to evaluate FI, with categories ranging from none, to mild, to moderate-to-severe. CVH was assessed through the “Life’s Essential 8” (LE8) framework established by the American Heart Association. This framework includes four health behaviors (diet quality, exercise, tobacco use, and sleep) and four biometric factors (body mass index, blood lipids, blood glucose, and blood pressure). Overall, CVH was scored on a scale from 0 to 100 and categorized into ideal (LE8 score: 80–100), intermediate (LE8 score: 50–79), and poor CVH (LE8 score: 0–49). Multivariate linear and logistic regression models were used to determine the association of FI with CVH status. Results: Within the sample, 3.7% and 0.8% experienced mild and moderate-to-severe FI, respectively. Compared with those without FI, individuals with mild (β: −2.92; 95% CI [confidence interval]: −4.37, −1.48) or moderate-to-severe (β: −7.71; 95% CI: −11.22, −4.20) FI had lower LE8 scores. Additionally, those with mild or moderate-to-severe FI were more likely to have poor CVH status (OR [odds ratio]: 2.14; 95% CI: 1.33, 3.66 for mild FI and OR: 4.83; 95% CI: 1.64, 14.17 for moderate-to-severe FI). Conclusions: FI is negatively associated with CVH in Korean adults. These findings underscore the need for comprehensive policy interventions to enhance food access and promote CVH in this at-risk population.

## 1. Introduction

Cardiovascular diseases (CVDs) represent the primary cause of death worldwide and in South Korea [1]. The increasing concern about CVDs highlights the necessity of identifying risk factors and implementing policies that promote cardiovascular health (CVH). As part of its efforts to reduce the health burdens associated with cardiovascular diseases (CVDs), the American Heart Association (AHA) established the framework of “Life’s Essential 8” (LE8) in 2022, an updated version of “Life’s Simple 7” (LS7) introduced in 2010 [2]. This framework is designed to quantify an individual’s cardiovascular health (CVH) and identify individuals at risk. It encompasses four health behaviors (diet, physical activity, tobacco use, and sleep) and four biometric factors (body mass index [BMI], blood glucose, blood lipids, and blood pressure). Recent research has consistently shown that a higher LE8 score is associated with lower risks of all-cause mortality and CVDs [3,4,5]. Furthermore, a systematic meta-analysis confirmed that LE8 is a reliable predictor of the onset and progression of CVDs, including ischemic heart disease and stroke [6].

Food insecurity (FI), defined as having limited access to nutritious and balanced food [7,8], affects approximately 4.3–4.5% of individuals in South Korea [9,10]. It has become a significant public health issue. Those affected by FI are more likely to report mental health problems, such as depression and stress [11]. Additionally, FI has been linked to higher risks of all-cause mortality and CVD-related deaths in a prospective cohort study [12]. Given the detrimental impact of FI on health, policy interventions are crucial to enhance food security among at-risk populations with low socio-economic status.

Some studies have examined the link between FI and CVH. For example, FI has been associated with poor CVH status as measured by the LS7 framework [13,14,15,16]. Likewise, recent research has shown that FI negatively correlates with the LE8 score among adolescents and adults in the United States (US) [17,18]. However, there are several gaps in the existing literature. Most notably, the majority of studies on this association have been conducted in the US, which limits the applicability of these findings to other regional contexts, including Asia. Food practices, prices, and policies regarding FI vary significantly by country, underscoring the need for further research in these diverse settings. Additionally, while the LE8 metric offers a more detailed assessment than the LS7 one, data on the relationship between FI and the LE8 score are still limited [17,18]. Therefore, this study aims to examine the relationship between FI and CVH status measured by the LE8 framework in a population-based sample of Korean adults.

## 2. Materials and Methods

### 2.1. Study Population

**Figure 1:**
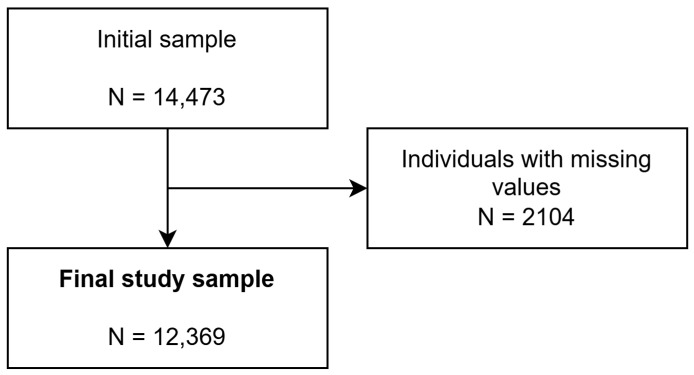
Flowchart of the selection process of the study sample.

This cross-sectional study employed secondary data analysis of the Korea National Health and Nutrition Examination Survey (KNHANES) data collected between 2019 and 2021. The KNHANES is a comprehensive health examination designed to assess the health behaviors and status of the Korean population. Utilizing multi-stage clustered probability sampling, the survey ensures a representative sample from various regions across South Korea. Initially, the survey included 14,473 adults aged ≥18 years. After the exclusion of 2104 individuals owing to missing data, 12,369 adults remained for analysis in our analysis (Figure 1). The survey was approved by the Institutional Review Board of the Korea Disease Control and Prevention Agency, with the following reference numbers: 2018-01-03-C-A, 2018-01-03-2C-A, and 2018-01-03-5C-A. Written informed consent was provided by all study participants.

### 2.2. FI

FI was assessed using the 18-item US Department of Agriculture Household Food Security Survey Module (HFSSM) that was adapted for the Korean context. The adaptation procedure and the validity of the Korean HFSSM have been documented in the previous literature [19,20]. The survey consists of 18 questions that gauge financial strain and access to food over the past year, with responses recorded as either “yes” or “no.” For households without children under 18, ten items were evaluated, while an additional eight items were included for households with children. Based on the cutoff criteria established in the literature [19,20], households were categorized as non-food insecure if they answered “yes” to fewer than three items (score of 0–2), regardless of child presence. Households with children scoring 3–7 were classified as mildly food insecure, and those scoring 8–18 were considered moderately to severely food insecure. For households without children, scores of 3–5 indicated mild FI, and scores of 6–10 indicated moderate-to-severe FI.

### 2.3. LE8

The LE8 framework comprises eight components, including four health behaviors (diet quality, the amount of physical activity per week, exposure to nicotine, and sleep duration) and four biometric factors (BMI, blood lipid level, blood glucose level, and blood pressure level). Each component is assigned a score ranging from 0 to 100, with higher scores indicating better CVH status. The composite LE8 score is derived by calculating the average of the individual scores across the eight components. The operationalization of each LE8 component in this study, based on the prior literature by the AHA, is detailed in Table S1 [2].

Dietary quality was determined using the validated Korean Healthy Eating Index, a tool designed to assess dietary quality among the Korean population [21]. The amount of physical activity per week was determined through the validated Korean Global Physical Activity Questionnaire [22], which measures activity across occupational, leisure-time, and transport-related domains. Total moderate-to-vigorous physical activity was then calculated. Nicotine exposure was categorized based on self-reported data regarding current and past smoking habits, including duration, electronic cigarette usage, and exposure to secondhand smoke within the household. Sleep health was assessed by average nightly sleep duration, derived from a self-reported questionnaire. BMI was calculated from objectively measured height and weight, with categorization following Asia–Pacific regional criteria (overweight: 23.0–24.9 kg/m^2^; obese: ≥25.0 kg/m^2^) [23]. Blood lipid levels were classified according to non-high-density lipoprotein (HDL) cholesterol, which was determined by subtracting HDL cholesterol from total cholesterol. Blood glucose was categorized based on glycated hemoglobin levels and reported diabetes history. Blood pressure was measured using a properly sized cuff.

Based on the cutoff points suggested by the AHA [2], individuals with a composite LE8 score of 80–100 were categorized as having “ideal CVH,” those with scores of 50–79 as having “intermediate CVH,” and those with scores of 0–49 as having “poor CVH.” Additionally, averages for the four health behaviors and four biometric factors were calculated separately and classified into ideal, intermediate, or poor categories using the same criteria.

### 2.4. Covariates

The following sociodemographic variables were adjusted in the regression models: sex (male, female); age groups (<30, 30–39, 40–49, 50–59, ≥60); education level (middle school or below, high school, college or above); income level (lowest, low, high, highest); marital status (married, unmarried, or other); and employment status (employed, unemployed). Total household income levels were grouped into four groups based on the quartile values for each year within the sample. All variables were self-reported.

### 2.5. Statistical Analysis

The features of the sample based on FI status were examined. We also assessed the prevalence of ideal, intermediate, and poor CVH across different study variables. To explore differences in the mean values of each component and the composite LE8 score across FI groups, we used analysis of covariance (ANOVA).

The relationship between FI and LE8 scores was analyzed using linear regression models. We fitted unadjusted, sex- and age-adjusted, and fully adjusted models, estimating beta coefficients (β) and 95% confidence intervals (CIs). Additionally, the association of FI with scores for health behaviors and biometric factors was evaluated using linear regression models. We further explored the relationship between FI and CVH categories using multinomial logistic regression, with ideal CVH status serving as the reference outcome. Odds ratios (ORs) and 95% CIs were computed. Statistical analyses and visualizations were conducted using R software (version 4.4.1). Survey weights were applied to the regression models to reflect the survey design, using the R package “survey.”

For the sensitivity analysis, we excluded individuals diagnosed with cerebrovascular disease, cardiovascular disease, or chronic kidney disease by a physician (n = 11,779).

## 3. Results

Table 1 presents the characteristics of the study sample (mean age: 51.8). Among the participants (n = 12,369), 3.7% (n = 458) experienced mild FI, and 0.8% (n = 101) experienced moderate-to-severe FI. Compared to individuals without FI, those experiencing FI were more likely to be older, have lower educational attainment, lower income levels, and be unmarried and unemployed.

**Figure 2:**
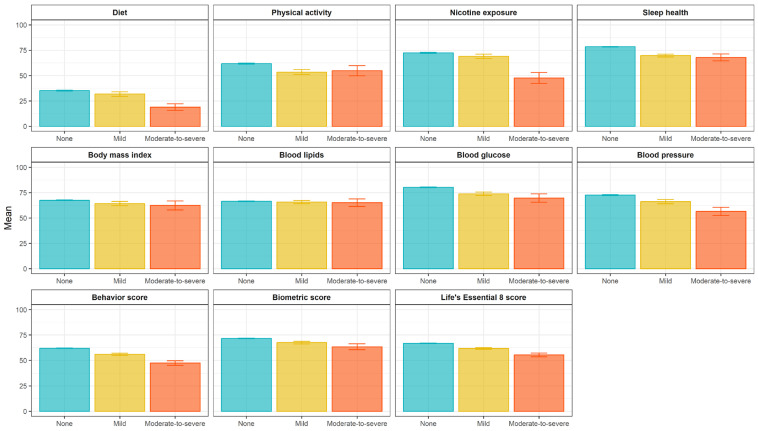
Weighted means of Life’s Essential 8 components according to food insecurity group. Notes: Error bars represent standard errors. The *p*-values from the covariance (ANOVA) analysis were <0.05 for all outcomes except blood lipids.

Figure 2 shows the mean values of each component and the composite LE8 score. The mean (standard deviation) LE8 score was 66.3 (13.8) across the sample. The composite LE8 scores averaged 66.6 for those without FI, 61.4 for those with mild FI, and 56.9 for those with moderate-to-severe FI. Similarly, the mean biometric scores were 70.3, 65.9, and 63.7, and the mean health behavior scores were 62.9, 56.8, and 50.1 for individuals without FI, with mild FI, and with moderate-to-severe FI, respectively.

Table S2 details the prevalence of CVH status according to the study sample. The prevalence of poor CVH was higher among individuals with mild or moderate-to-severe FI, men, older adults, those with lower educational levels, and those with lower income levels.

Tables S3 and S4 indicate that the relationship between LE8 and CVH was consistently observed after excluding individuals with cerebrovascular, cardiovascular, or chronic kidney disease.

Table 2 presents the association of FI with LE8 scores, along with its subcomponents. Compared to those without FI, the LE8 score was lower among those with mild (adjusted *β*: −2.92; 95% CI: −4.37, −1.48) and moderate-to-severe FI (adjusted *β*: −7.71; 95% CI: −11.22, −4.20). Similarly, the behavior score was lower among those with mild (adjusted *β*: −4.26; 95% CI: −6.60, −1.91) and moderate-to-severe FI (adjusted β: −10.13; 95% CI: −14.59, −5.67). Individuals with moderate-to-severe FI also had a lower biometric score compared to those with less severe FI (adjusted *β*: −5.29; 95% CI: −10.38, −0.20).

Table 3 also shows the association of FI with CVH categories. Compared to those without FI, individuals with mild (OR: 2.14; 95% CI: 1.33, 3.66) or moderate-to-severe FI (OR: 4.83; 95% CI: 1.64, 14.17) were more likely to have poor CVH. Similarly, individuals with mild (OR: 1.88; 95% CI: 1.26, 2.79) or moderate-to-severe FI (OR: 5.33; 95% CI: 2.11, 13.43) were more likely to have poor CVH in behavioral factors. Those with moderate-to-severe FI were more likely to exhibit poor CVH in biometric factors compared to those without FI (OR: 2.53; 95% CI: 1.26, 5.07).

## 4. Discussion

The findings of our study indicate that FI is inversely associated with the LE8 score, even after adjusting for sociodemographic factors. Specifically, FI was strongly linked to undesirable health behaviors. Specifically, FI was associated with poor diet quality, nicotine exposure, and short sleep duration, and was also related to elevated blood pressure. Although the associations with other factors were not statistically significant, they generally showed negative correlations. Therefore, our results suggest that policy efforts are crucial to improving food security and promoting CVH among vulnerable populations.

The results of this study align with those of previous research, which has shown a link between FI and compromised CVH. For example, in the US, individuals with FI were less likely to achieve ideal CVH status [16]. Furthermore, FI has been linked to multiple indicators of CVH risk as measured by the LE8 framework among U.S. adults and adolescents [17,18]. However, no significant correlation was found between FI and LS7 scores among obese patients in the U.S [13]. While previous population-based studies have identified a connection between FI and poor CVH, this evidence was primarily observed within the US population. Thus, the present study expands the existing literature by revealing that this relationship also holds in a nationally representative sample of Korean adults, which enhances its generalizability.

Various mechanisms may explain the association of FI with poor CVH. First, FI hampers the ability to consume a balanced diet, leading to an inadequate intake of nutrients. This nutritional deficiency can reduce dietary quality and elevate risk factors for CVH, such as blood glucose, blood pressure, and cholesterol levels [10,24,25]. Second, the psychological stress associated with FI may drive individuals toward unhealthy behaviors and contribute to adverse health conditions. For example, individuals facing FI may experience anxiety and stress about obtaining food, which can lead to sleep disturbances or a reliance on tobacco products as coping mechanisms [26]. Additionally, the physiological response to chronic stress exposure can lead to dysregulation, such as elevated blood pressure, and negatively impact overall health [27,28]. Third, the possibility of reverse causality should be considered, where poor health behaviors or conditions may contribute to FI. For instance, individuals with nicotine dependence might spend a significant portion of their income on tobacco products, which can limit the financial resources available for food [29]. Moreover, individuals with poor CVH status may face high medical costs, further reducing their financial capacity to secure food [30].

This study highlights the need for policy measures aimed at improving food accessibility to promote public health. Various approaches are required to enhance the multifaceted aspects of food accessibility, including availability, affordability, and dietary quality [31]. A recent study indicated that financial and food assistance, as well as nutritional education, may contribute to enhancing food security [32]. These policies may be most effective when implemented in combination, warranting a comprehensive policy approach [32].

This study has some limitations. First, the cross-sectional design limits the ability to assert causal effects, and the potential for reverse causation should be carefully considered in the interpretation of results. Thus, future research should employ longitudinal analyses to clarify the temporal relationship between FI and CVH status. Second, as this is an observational study, the possibility of unmeasured confounders exists. For example, we were unable to account for factors such as genetic predisposition, a family history of CVDs, working conditions [33], and social support due to the unavailability of this data. Third, while biometric factors were objectively measured by health professionals, health behaviors were self-reported by participants. This could introduce measurement errors, including recall bias and misclassification bias [34]. For instance, social desirability bias, where participants may overreport their physical activity and underreport their tobacco use, should also be considered.

This study is strengthened by its focus on a nationally representative sample of Korean adults, a population that has been relatively understudied in the literature. Moreover, the use of comprehensive survey data from the KNHANES enabled the rigorous application of the LE8 scoring system while ensuring it was adapted to the Korean context. While the connection between poor nutrition and CVD is not entirely new, the novelty of our study lies in examining the association between FI and CVH in the Korean population, or more broadly in an Asian population, given that the majority of existing research has been conducted among U.S. populations [16,17,18]. In addition, this study newly highlights that FI is associated not only with biometric health factors but also with various health behaviors linked to CVD risk factors, underscoring the potential need for health promotion efforts targeting individuals with food security to prevent CVD. Finally, the associations between food insecurity and various CVH factors provide insight into the mechanisms by which individuals experiencing food insecurity may be at increased risk for the onset of CVD.

## 5. Conclusions

This study found that FI is inversely associated with CVH, as assessed by the LE8 framework. Additionally, FI is closely linked to undesirable health behaviors that contribute to CVH risk factors. Although future longitudinal research is required to clarify the causal relationships, this study underscores the need for comprehensive policy measures aimed at improving food accessibility and promoting CVH among this vulnerable population.

## Figures and Tables

**Table 1:** Baseline characteristics of the study sample according to food insecurity.

	Overall	Food Insecurity		
		None	Mild	Moderate-to-Severe
	N = 12,369	N = 11,809	N = 459	N = 101
Sex				
Male	5259 (42.5)	5041 (42.7)	169 (36.8)	49 (48.5)
Female	7110 (57.5)	6768 (57.3)	290 (63.2)	52 (51.5)
Age (categorical)				
<30	1553 (12.6)	1500 (12.7)	42 (9.2)	11 (10.9)
30–39	1706 (13.8)	1666 (14.1)	27 (5.9)	13 (12.9)
40–49	2236 (18.1)	2156 (18.3)	72 (15.7)	8 (7.9)
50–59	2325 (18.8)	2233 (18.9)	71 (15.5)	21 (20.8)
≥60	4549 (36.8)	4254 (36.0)	247 (53.8)	48 (47.5)
Age (continuous)				
Mean (SD)	51.8 (16.8)	51.6 (16.7)	57.8 (17.1)	55.7 (17.3)
Education level				
Middle school or below	3365 (27.2)	3079 (26.1)	236 (51.4)	50 (49.5)
High school	4147 (33.5)	3958 (33.5)	150 (32.7)	39 (38.6)
College or above	4857 (39.3)	4772 (40.4)	73 (15.9)	12 (11.9)
Income level				
Lowest	2223 (18.0)	1918 (16.2)	242 (52.7)	63 (62.4)
Low	3037 (24.6)	2881 (24.4)	126 (27.5)	30 (29.7)
High	3366 (27.2)	3289 (27.9)	71 (15.5)	6 (5.9)
Highest	3743 (30.3)	3721 (31.5)	20 (4.4)	2 (2.0)
Marital status				
Married	8327 (67.3)	8059 (68.2)	239 (52.1)	29 (28.7)
Unmarried/others	4042 (32.7)	3750 (31.8)	220 (47.9)	72 (71.3)
Employment status				
Employed	7425 (60.0)	7159 (60.6)	218 (47.5)	48 (47.5)
Unemployed	4944 (40.0)	4650 (39.4)	241 (52.5)	53 (52.5)
Korean Healthy eating index				
Mean (SD)	60.3 (13.3)	60.5 (13.2)	58.7 (13.3)	52.9 (15.8)
Total physical activity (min/wk)				
Mean (SD)	210.3 (346.8)	211.7 (346.5)	176.9 (344.4)	198.5 (382.7)
Smoking status				
Never smoker	7590 (61.4)	7272 (61.6)	271 (59.0)	47 (46.5)
Past smoker	2815 (22.8)	2698 (22.8)	102 (22.2)	15 (14.9)
Current smoker	1964 (15.9)	1839 (15.6)	86 (18.7)	39 (38.6)
Sleep duration (h/day)				
Mean (SD)	7.4 (1.7)	7.4 (1.7)	7.2 (2.2)	6.9 (2.0)
Health behavior score				
Mean (SD)	62.5 (18.9)	62.9 (18.8)	56.8 (19.5)	50.1 (20.4)
Body mass index (kg/m^2^)				
Mean (SD)	24.0 (3.6)	24.0 (3.6)	24.5 (4.2)	24.9 (4.1)
Non-HDL cholesterol				
Mean (SD)	138.3 (37.8)	138.5 (37.7)	135.8 (38.7)	133.5 (40.6)
HbA1c (%)				
Mean (SD)	5.8 (0.8)	5.8 (0.8)	6.0 (1.0)	6.1 (1.3)
Systolic blood pressure (mmHg)				
Mean (SD)	118.7 (16.5)	118.6 (16.5)	121.5 (17.0)	126.0 (18.3)
Diastolic blood pressure (mmHg)				
Mean (SD)	74.8 (9.8)	74.8 (9.8)	74.1 (10.0)	77.1 (10.2)
Biometric score				
Mean (SD)	70.1 (18.8)	70.3 (18.7)	65.9 (19.1)	63.7 (19.8)
Life’s Essential 8				
Mean (SD)	66.3 (13.8)	66.6 (13.7)	61.4 (13.7)	56.9 (14.7)

SD, standard deviation; wk, week. Values are presented as n (column %) for categorical variables.

**Table 2:** Association between food insecurity and LE8 and its subcomponent scores.

	Model 1	Model 2	Model 3
	*β* (95% CI)	*β* (95% CI)	*β* (95% CI)
**LE8 score**			
Food insecurity			
None	Reference	Reference	Reference
Mild	−5.10 (−6.74, −3.46)	−4.87 (−6.30, −3.44)	−2.92 (−4.37, −1.48)
Moderate-to-severe	−11.47 (−15.28, −7.67)	−10.67 (−14.20, −7.14)	−7.71 (−11.22, −4.20)
**Diet score**			
Food insecurity			
None	Reference	Reference	Reference
Mild	−3.41 (−7.81, 0.99)	−7.36 (−11.94, −2.78)	−4.91 (−9.48, −0.34)
Moderate-to-severe	−16.30 (−22.59, −10.01)	−17.82 (−24.08, −11.56)	−14.02 (−20.21, −7.83)
**Physical activity score**			
Food insecurity			
None	Reference	Reference	Reference
Mild	−8.44 (−13.33, −3.54)	−6.21 (−11.15, −1.26)	−2.37 (−7.52, 2.79)
Moderate-to-severe	−7.05 (−16.82, 2.72)	−6.14 (−15.32, 3.04)	−0.85 (−10.18, 8.47)
**Nicotine exposure score**			
Food insecurity			
None	Reference	Reference	Reference
Mild	−3.45 (−7.88, 0.99)	−7.02 (−11.10, −2.94)	−4.63 (−8.89, −0.37)
Moderate-to-severe	−24.74 (−35.42, −14.07)	−23.59 (−34.10, −13.09)	−19.50 (−29.73, −9.27)
**Sleep health score**			
Food insecurity			
None	Reference	Reference	Reference
Mild	−8.67 (−11.53, −5.81)	−7.72 (−10.52, −4.92)	−5.12 (−7.99, −2.25)
Moderate-to-severe	−10.49 (−17.24, −3.74)	−10.04 (−16.63, −3.46)	−6.15 (−12.61, 0.30)
**Health behavior score**			
Food insecurity			
None	Reference	Reference	Reference
Mild	−5.99 (−8.30, −3.68)	−7.08 (−9.32, −4.84)	−4.26 (−6.60, −1.91)
Moderate-to-severe	−14.64 (−19.07, −10.21)	−14.40 (−18.97, −9.83)	−10.13 (−14.59, −5.67)
**Body mass index score**			
Food insecurity			
None	Reference	Reference	Reference
Mild	−3.23 (−7.46, 0.99)	−3.60 (−7.67, 0.47)	−2.27 (−6.37, 1.83)
Moderate-to-severe	−5.08 (−13.81, 3.66)	−4.22 (−12.85, 4.41)	−2.44 (−11.20, 6.31)
**Blood lipids score**			
Food insecurity			
None	Reference	Reference	Reference
Mild	−0.76 (−4.01, 2.49)	−0.52 (−3.55, 2.52)	−1.35 (−4.41, 1.71)
Moderate-to-severe	−1.44 (−8.82, 5.95)	−1.20 (−8.30, 5.89)	−2.23 (−9.28, 4.82)
**Blood glucose score**			
Food insecurity			
None	Reference	Reference	Reference
Mild	−6.39 (−9.83, −2.96)	−3.01 (−6.03, 0.02)	−1.94 (−5.00, 1.11)
Moderate-to-severe	−10.57 (−18.69, −2.46)	−8.47 (−15.65, −1.29)	−6.89 (−14.09, 0.32)
**Blood pressure score**			
Food insecurity			
None	Reference	Reference	Reference
Mild	−6.44 (−10.70, −2.18)	−3.51 (−7.28, 0.27)	−0.81 (−4.62, 3.01)
Moderate-to-severe	−16.12 (−24.07, −8.17)	−13.84 (−20.93, −6.74)	−9.61 (−16.86, −2.36)
**Biometric factor score**			
Food insecurity			
None	Reference	Reference	Reference
Mild	−4.21 (−6.74, −1.68)	−2.66 (−4.79, −0.53)	−1.59 (−3.75, 0.59)
Moderate-to-severe	−8.30 (−13.99, −2.61)	−6.93 (−11.91, −1.95)	−5.29 (−10.38, −0.20)

CI, confidence interval; LE8, Life’s Essential 8. Model 1: unadjusted model. Model 2: Model 1 + sex + age. Model 3: Model 2 + education + income + marital status + employment status.

**Table 3:** Association between food insecurity and cardiovascular health category on multinomial regression models.

	CVH Categories (Reference Outcome: Ideal CVH)	
	Intermediate CVH	Poor CVH
	OR (95% CI)	OR (95% CI)
**LE8 score**		
Food insecurity		
None	Reference	Reference
Mild	1.63 (1.09, 2.43)	2.14 (1.33, 3.66)
Moderate-to-severe	1.44 (0.54, 3.80)	4.83 (1.64, 14.17)
**Diet score**		
Food insecurity		
None	Reference	Reference
Mild	1.20 (0.80, 1.79)	1.47 (0.94, 2.30)
Moderate-to-severe	1.60 (0.71, 3.62)	3.21 (1.47, 7.02)
**Physical activity score**		
Food insecurity		
None	Reference	Reference
Mild	1.25 (0.83, 1.90)	1.15 (0.90, 1.49)
Moderate-to-severe	2.14 (1.01, 4.54)	1.04 (0.65, 1.67)
**Nicotine exposure score**		
Food insecurity		
None	Reference	Reference
Mild	1.13 (0.79, 1.61)	1.54 (1.06, 2.24)
Moderate-to-severe	0.91 (0.38, 2.14)	3.43 (1.68, 7.00)
**Sleep health score**		
Food insecurity		
None	Reference	Reference
Mild	1.39 (1.04, 1.85)	1.55 (1.23, 1.94)
Moderate-to-severe	0.78 (0.40, 2.53)	1.65 (1.05, 2.61)
**Health behavior score**		
Food insecurity		
None	Reference	Reference
Mild	1.48 (1.04, 2.12)	1.88 (1.26, 2.79)
Moderate-to-severe	3.46 (1.52, 7.90)	5.33 (2.11, 13.43)
**Body mass index score**		
Food insecurity		
None	Reference	Reference
Mild	1.34 (0.97, 1.83)	1.14 (0.84, 2.56)
Moderate-to-severe	0.71 (0.37, 1.36)	1.07 (0.60, 1.91)
**Blood lipids score**		
Food insecurity		
None	Reference	Reference
Mild	1.24 (0.94, 1.63)	1.03 (0.80, 1.34)
Moderate-to-severe	1.07 (0.60, 1.89)	1.00 (0.55, 1.79)
**Blood glucose score**		
Food insecurity		
None	Reference	Reference
Mild	1.08 (0.82, 1.43)	1.21 (0.83, 1.77)
Moderate-to-severe	0.62 (0.34, 1.14)	2.39 (1.19, 4.81)
**Blood pressure score**		
Food insecurity		
None	Reference	Reference
Mild	1.07 (0.77, 1.48)	1.07 (0.78, 1.46)
Moderate-to-severe	1.74 (0.95, 3.18)	2.18 (1.15, 4.14)
**Biometric factor score**		
Food insecurity		
None	Reference	Reference
Mild	0.95 (0.71, 1.27)	1.26 (0.86, 1.82)
Moderate-to-severe	0.94 (0.52, 1.68)	2.53 (1.26, 5.07)

OR, odds ratio; CI, confidence interval; CVH, cardiovascular health; LE8, Life’s Essential 8. All models were adjusted for sex, age, education, income, marital status, and employment status.

## Data Availability

Data are available at https://knhanes.kdca.go.kr/knhanes (accessed on 1 March 2025).

## Supplementary Materials

The following supporting information can be downloaded at https://www.mdpi.com/article/10.3390/nu17132148/s1: Table S1. Operationalization of Life’s Essential 8 score; Table S2. Distribution of cardiovascular health status according to study variables; Table S3. Association between food insecurity and LE8 and its subcomponent scores; Table S4. Association between food insecurity and cardiovascular health category on multinomial regression models.
