# Supplementary material for: Association Between Food Insecurity and Poor Cardiovascular Health Assessed by the Life’s Essential 8 Metric: A Population-Based Study of Korean Adults
Source: Nutrients. 2025 Jun 27;17(13):2148. doi: 10.3390/nu17132148 (PMC12251093; doi:10.3390/nu17132148)
Supplement: Supplementary file 1 [file nutrients-17-02148-s001.zip › nutrients-3669303-supplementary.pdf]

Table S1. Operationalization of Life's Essential 8 score

Health factors	Measurement	Scoring system (categorization)
**1. Diet**	Korea Healthy Eating Index (range: 0–100)	100: ≥95th percentile; HEI: 81.2–100<br>80: 75–94th percentile; HEI: 70.1–82.2<br>50: 50–74th percentile; HEI: 60.8–70.1<br>25: 25–49th percentile; HEI: 51.2–60.8<br>0: <25th percentile; <51.2
**2. Physical activity**	Korean Global Physical Activity Questionnaire (unit: MVPA per week)	100: ≥150 MVPA/week<br>90: 120–149 MVPA/week<br>80: 90–119 MVPA/week<br>60: 60–89 MVPA/week<br>40: 30–59 MVPA/week<br>20: 1–29 MVPA/week<br>0: 0 MVPA/week
**3. Nicotine exposure**[a]	Self-reported questionnaire: current smoking status, past smoking history, use of e-cigarettes, exposure to household indoor smoking	100: never smoker<br>75: former smoker, quit ≥5 year<br>50: former smoker, quit 1–<5 year<br>25: former smoker, quit <1 y<br>25: current use of e-cigarettes<br>0: current smoker
**4. Sleep health**	Self-reported questionnaire: average hours of sleep per night	100: 7–<9 h<br>75: 9–<10 h<br>50: 6–<7 h<br>40: 5–<6 h<br>40: ≥10 h<br>20: 4–<5 h<br>0: <4 h
**5. BMI**	Objective measurement of body weight and heights	BMI<br>100: <23 kg/m^2<br>75: 23.0–24.9 kg/m^2<br>30: 25.0–29.9 kg/m^2<br>15: 30.0–34.9 kg/m^2<br>0: ≥35 kg/m^2
**6. Blood lipids**[b]	Total cholesterol, HDL cholesterol, current use of lipid-lowering agents	Non-HDL cholesterol<br>100: <130 mg/dL<br>60: 130–159 mg/dL<br>40: 160–189 mg/dL<br>20: 190–219 mg/dL<br>0: ≥220 mg/dL
**7. Blood glucose**	HbA1c, current use of oral anti-diabetic medications or insulin	100: No diabetes history and HbA1c <5.7%<br>60: No diabetes history and HbA1c 5.7–6.4%<br>40: Diabetes with HbA1c <7.0%<br>30: Diabetes with HbA1c 7.0–7.9%<br>20: Diabetes with HbA1c 8.0–8.9%<br>10: Diabetes with HbA1c 9.0–9.9%<br>0: Diabetes with HbA1c ≥10.0%
**8. BP**[b]	Approximately sized BP cuff, current use of anti-hypertensive drug	Systolic BP and diastolic BP<br>100: <120/<80 mmHg<br>75: 120–129/<80 mmHg<br>50: 130–139 or 80–89 mmHg<br>25: 140–159 or 90–99 mmHg<br>0: ≥160 or ≥100 mmHg

**Total LE8 score**	Range: 0–100	80–100: High CVH<br>50–79: Intermediate CVH<br>0–49: Low CVH

[a] Subtract 20 points if exposed to indoor smoking in households

[b] Subtract 20 points if drug-treated level

BMI, body mass index; BP, blood pressure; HDL cholesterol, High-density lipoprotein cholesterol; MVPA, moderate-to-vigorous physical activity; e-cigarette, electronic cigarette; HbA1c, hemoglobin A1c

Table S2 Distribution of cardiovascular health status according to study variables

	CVH status (LE8 score)		
	**Ideal CVH (80–100)**	**Intermediate CVH (50–79)**	**Poor CVH (0–49)**
Food insecurity			
None	2,201 (18.6)	8,235 (69.7)	1,373 (11.6)
Mild	44 (9.6)	328 (71.5)	87 (19.0)
Moderate-to-severe	9 (8.9)	55 (54.5)	37 (36.6)
Sex			
Male	511 (9.7)	3,794 (72.1)	954 (18.1)
Female	1,743 (24.5)	4,824 (67.8)	543 (7.6)
Age			
<30	495 (31.9)	986 (63.5)	72 (4.6)
30–39	464 (27.2)	1,075 (63.0)	167 (9.8)
40–49	512 (22.9)	1,442 (64.5)	282 (12.6)
50–59	343 (14.8)	1,630 (70.1)	352 (15.1)
≥60	440 (9.7)	3,485 (76.6)	624 (13.7)
Education level			
Middle school or below	250 (7.4)	2,530 (75.2)	585 (17.4)
High school	761 (18.4)	2,891 (69.7)	495 (11.9)
College or above	1,243 (25.6)	3,197 (65.8)	417 (8.6)
Income level			
Lowest	207 (9.3)	1,621 (72.9)	395 (17.8)
Low	481 (15.8)	2,163 (71.2)	393 (12.9)
High	693 (20.6)	2,276 (67.6)	397 (11.8)
Highest	873 (23.3)	2,558 (68.3)	312 (8.3)
Marital status			
Married	1,470 (17.7)	5,884 (70.7)	973 (11.7)
Unmarried/others	784 (19.4)	2,734 (67.6)	524 (13.0)
Employment status			
Employed	1,342 (18.1)	5,155 (69.4)	928 (12.5)
Unemployed	912 (18.4)	3,463 (70.0)	569 (11.5)

Values are presented as n (column %).

CVH, cardiovascular health; LE8, Life's Essential 8

Ideal CVH: LE8 80-100; Intermediate CVH: LE8 50-79; Poor CVH: LE8 0-49

Table S3 Association between food insecurity and LE8 and its subcomponent scores

	**Model 1**	**Model 2**	**Model 3**
	***β* (95% CI)**	***β* (95% CI)**	***β* (95% CI)**
**LE8 score**			
Food insecurity			
None	Reference	Reference	Reference
Mild	-4.90 (-6.67, -3.12)	-4.90 (-6.43, -3.37)	-2.94 (-4.48, -1.39)
Moderate-to-severe	-10.98 (-14.90, -7.06)	-10.41 (-14.06, -6.76)	-7.69 (-11.35, -4.03)
**Health behavior score**			
Food insecurity			
None	Reference	Reference	Reference
Mild	-5.61 (-8.06, -3.15)	-6.85 (-9.25, -4.45)	-4.01 (-6.53, -1.49)
Moderate-to-severe	-13.72 (-18.32, -9.12)	-13.59 (-18.43, -8.76)	-9.63 (-14.38, -4.88)
**Biometric factor score**			
Food insecurity			
None	Reference	Reference	Reference
Mild	-4.19 (-6.90, -1.47)	-2.95 (-5.21, -0.70)	-1.86 (-4.14, 0.41)
Moderate-to-severe	-8.24 (-14.34, -2.15)	-7.23 (-12.47, -1.98)	-5.75 (-11.11, -0.39)

CI, confidence interval; LE8, Life's Essential 8
Model 1: Unadjusted model
Model 2: Model 1 + sex + age
Model 3: Model 2 + education + income + marital status + employment status

Table S4 Association between food insecurity and cardiovascular health category on multinomial regression models

	CVH categories (reference outcome: ideal CVH)	
	**Intermediate CVH**	**Poor CVH**
	**OR (95% CI)**	**OR (95% CI)**
**LE8 category**		
Food insecurity		
None	Reference	Reference
Mild	1.61 (1.07, 2.41)	2.15 (1.30, 3.54)
Moderate-to-severe	1.39 (0.52, 3.74)	4.89 (1.65, 14.55)
**Health behavior category**		
Food insecurity		
None	Reference	Reference
Mild	1.43 (0.99, 2.07)	1.77 (1.17, 2.69)
Moderate-to-severe	3.66 (1.51, 8.85)	5.38 (1.97, 14.71)
**Biometric factor category**		
Food insecurity		
None	Reference	Reference
Mild	0.97 (0.72, 1.30)	1.30 (0.88, 1.92)
Moderate-to-severe	0.96 (0.52, 1.78)	2.67 (1.31, 5.44)

OR, odds ratio; CI, confidence interval; CVH, cardiovascular health; LE8, Life's Essential 8
All models were adjusted for sex, age, education, income, marital status, and employment status
